# Supplementary material for: Structural insight into the distinct regulatory mechanism of the HEPN–MNT toxin-antitoxin system in Legionella pneumophila
Source: Nat Commun. 2024 Nov 24;15:10188. doi: 10.1038/s41467-024-54551-0 (PMC11586414; doi:10.1038/s41467-024-54551-0)
Supplement: Supplementary file 1 — Supplementary Information [file 41467_2024_54551_MOESM1_ESM.pdf]

Supplementary information

Structural insight into the distinct regulatory mechanism of the HEPN–MNT toxin–antitoxin system in *Legionella pneumophila*

Authors: Chenglong Jin[1,2,8], Cha-Hee Jeon[1,3,8], Heung Wan Kim[1], Jin Mo Kang[1,3], Yuri Choi[4], Sung-Min Kang[5], Hyung Ho Lee[4], Do-Hee Kim[6,9], Byung Woo Han[1,3,9] and Bong-Jin Lee[2,7,9]

Affiliations

[1] The Research Institute of Pharmaceutical Sciences, College of Pharmacy, Seoul National University, Seoul 08826, Republic of Korea

[2] MasterMediTech, Seoul 07793, Republic of Korea

[3] Natural Products Research Institute, College of Pharmacy, Seoul National University, Seoul 08826, Republic of Korea

[4] Department of Chemistry, College of Natural Sciences, Seoul National University, Seoul 08826, Republic of Korea

[5] College of Pharmacy, Duksung Women’s University, Seoul 01369, Republic of Korea

[6] Research Institute of Pharmaceutical Sciences, College of Pharmacy, Sookmyung Women's University, Seoul, 04310, Republic of Korea

[7] College of Pharmacy, Ajou University, Suwon 16499, Republic of Korea

[8] These authors contributed equally: Chenglong Jin and Cha-Hee Jeon

[9] These authors jointly supervised this work: Do-Hee Kim, Byung Woo Han and Bong-Jin Lee

Corresponding authors:

- Do-Hee Kim (E-mail: dohee.kim@sookmyung.ac.kr)
- Byung Woo Han (E-mail: bwhan@snu.ac.kr)
- Bong-Jin Lee (E-mail: lbj@nmr.snu.ac.kr)

This file contains:

- Supplementary Figure 1–11
- Supplementary Table 1–4

Supplementary Figure 1

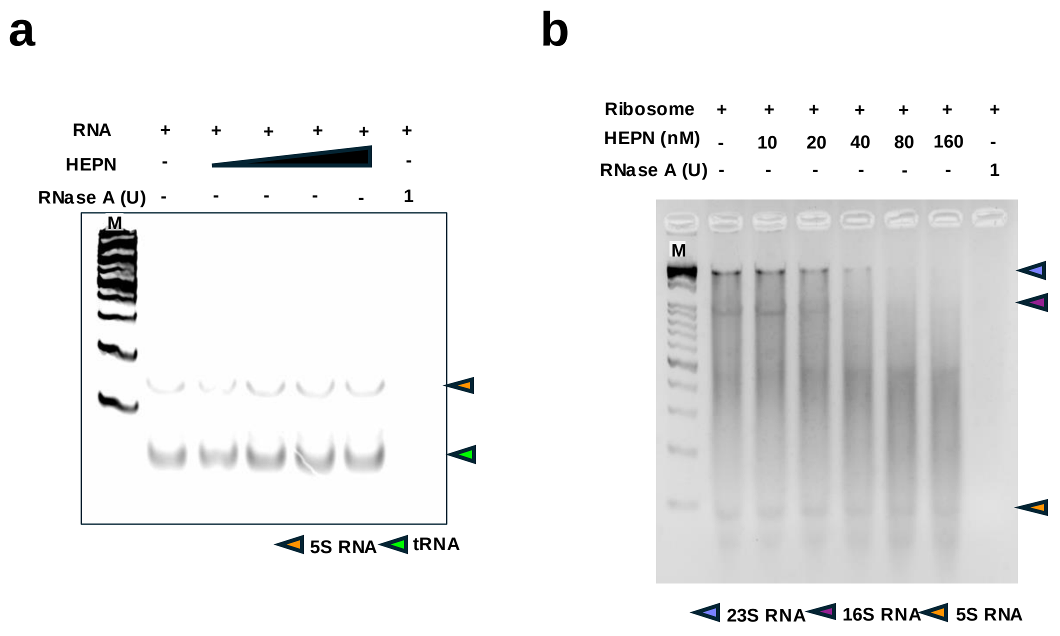

Supplementary Fig. 1. *In Vitro* RNase Assay

a A 12% polyacrylamide gel shows that dimeric HEPN^{Lpg} does not digest 5S rRNA and tRNA. Dimeric HEPN^{Lpg} was tested at varying concentrations (20 nM–160 nM) in this assay. **b** A 1.8% agarose gel shows that dimeric HEPN^{Lpg} cleaves 23S rRNA and 16S rRNA present in an intact 70S ribosome. The 70S ribosome from an *E. coli* B strain was used for this assay. RNase A (1 U) was used as a positive control.

Supplementary Figure 2

a

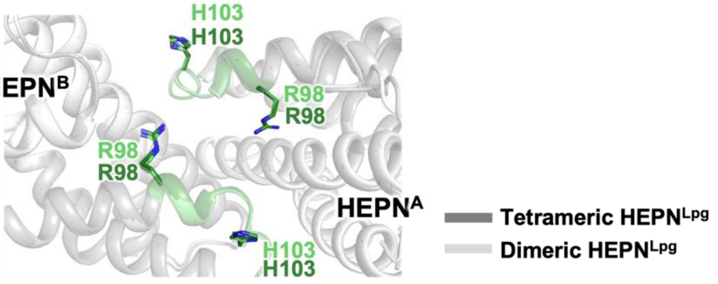

b

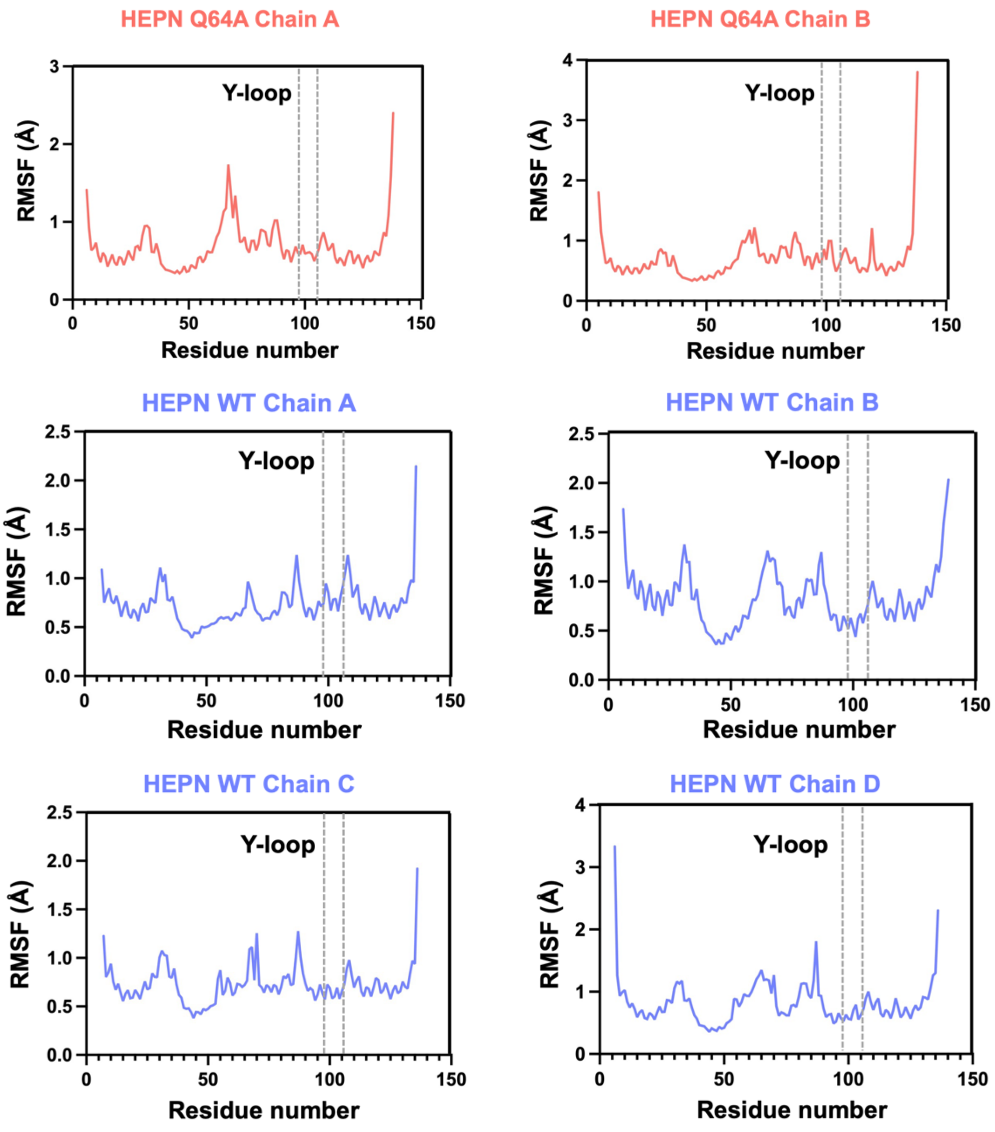

Supplementary Fig. 2. Structural comparison of the Y-loop region of HEPN^{Lpg} in two oligomeric states. **a** Structural alignment of the HEPN^{Lpg} dimer from the crystal structures of tetrameric and dimeric HEPN^{Lpg}. The Y-loop region from tetrameric HEPN^{Lpg} and dimeric HEPN^{Lpg} is highlighted in dark green and lime, respectively. **b** RMSF analysis of HEPN^{Lpg} in two oligomeric states. The RMSF values of the Cα atoms are plotted as a function of residues for chains A/B of dimeric HEPN^{Lpg} and chains A/B/C/D of tetrameric HEPN^{Lpg}. The residues located in the Y-loop regions are indicated with dashed boxes.

Supplementary Figure 3

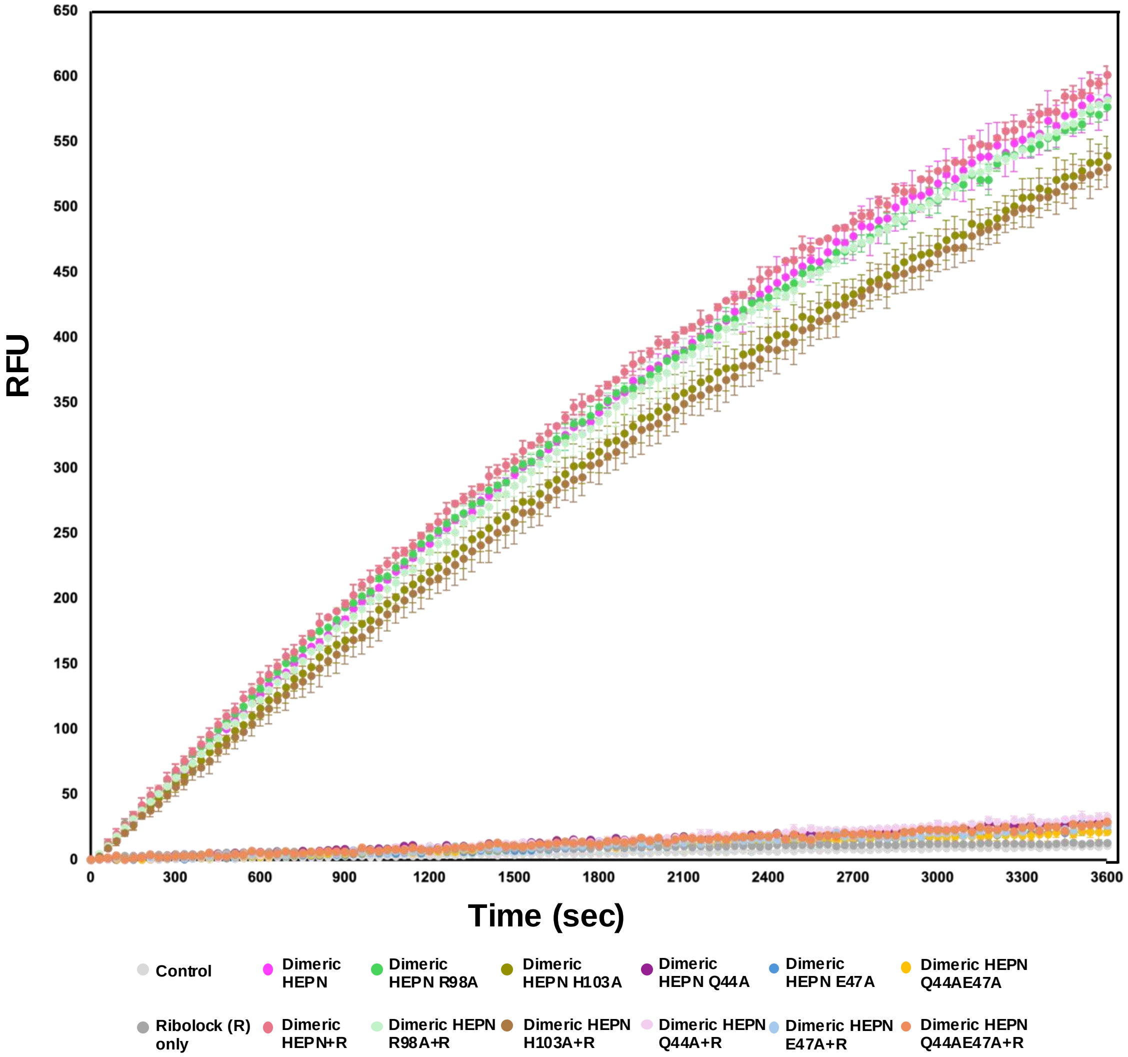

Supplementary Fig. 3. *In vitro* RNase activity assay

Fluorescence kinetics measured for dimeric HEPN^{Lpg} and its mutants with and without treatment of Ribolock RNase inhibitor. Minimal RFU variation between Ribolock-treated and untreated samples confirms the specificity of RNase activity, ruling out contamination by external RNases. The data are presented as the average of two independent replicates with standard deviation (±SD).

Supplementary Figure 4

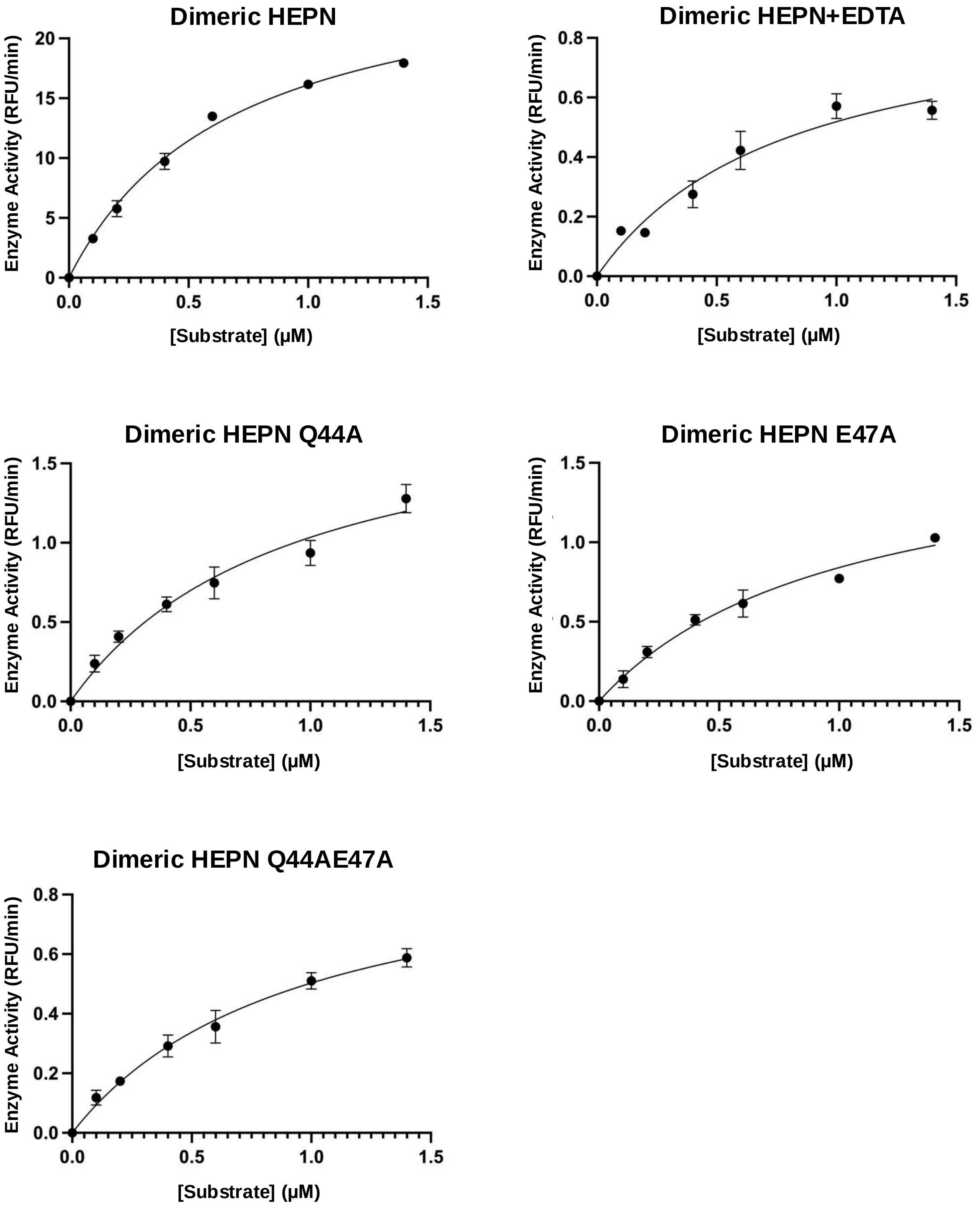

Supplementary Fig. 4. Plot of *v* versus [S] according to the Michaelis–Menten equation.

Dimeric $HEPN^{Lpg}$, EDTA-treated dimeric $HEPN^{Lpg}$, dimeric $HEPN^{Lpg}$ Q44A, dimeric $HEPN^{Lpg}$ E47A and dimeric $HEPN^{Lpg}$ Q44AE47A were subjected to *in vitro* RNase assays. The resulting data were processed using the Michaelis–Menten equation, and the results are presented in Supplementary Table 2.

Supplementary Figure 5

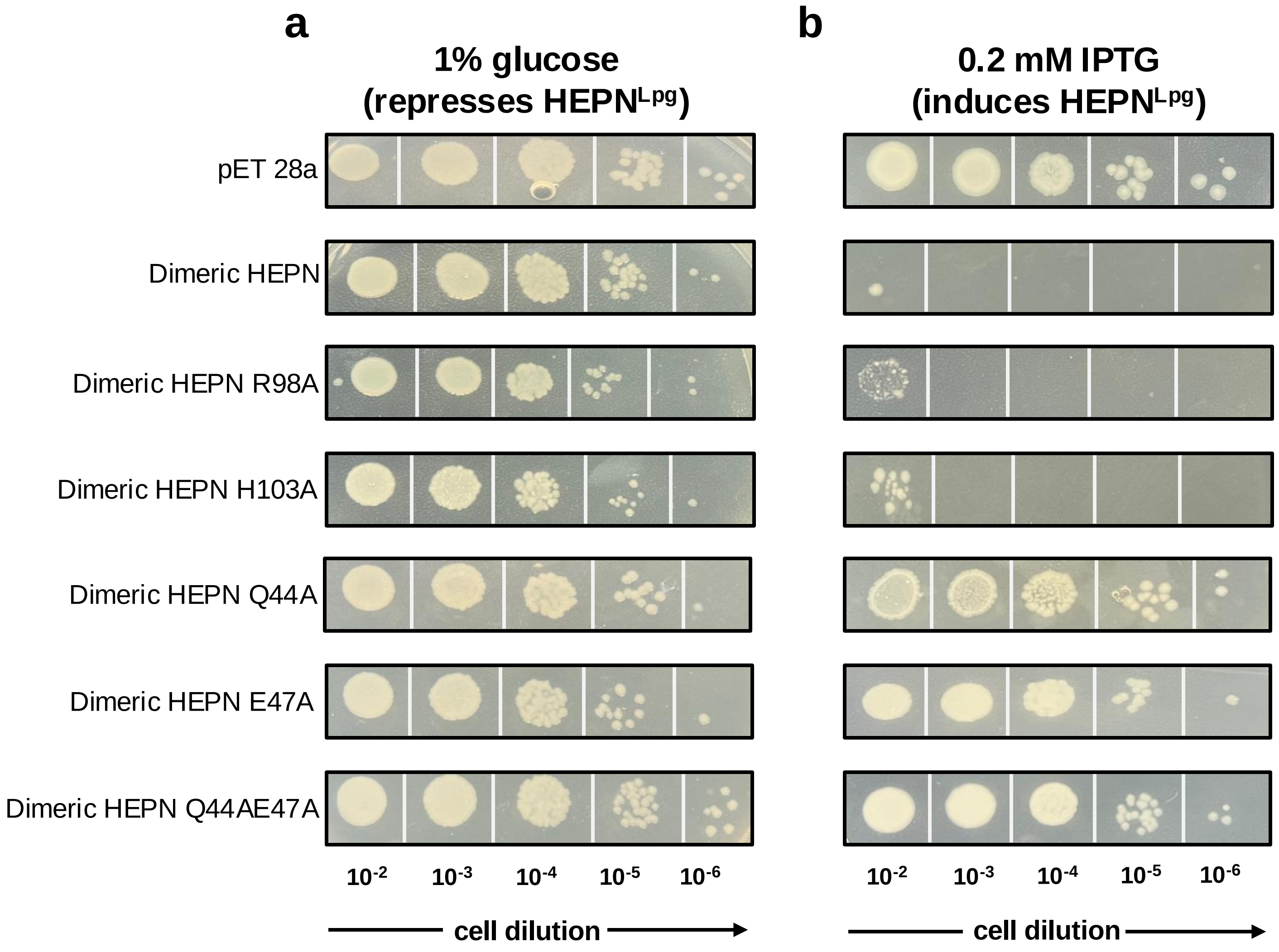

Supplementary Fig. 5. *In vivo* spot-plating assay

E. Coli Rosetta2 (DE3) cells harboring pET 28a vector with each HEPN^{Lpg} variant as indicated. The plates contains 1% glucose to repress background expression from T7 promotor (**a**) and 0.2 mM IPTG to induce HEPN^{Lpg} expression (**b**).

Supplementary Figure 6

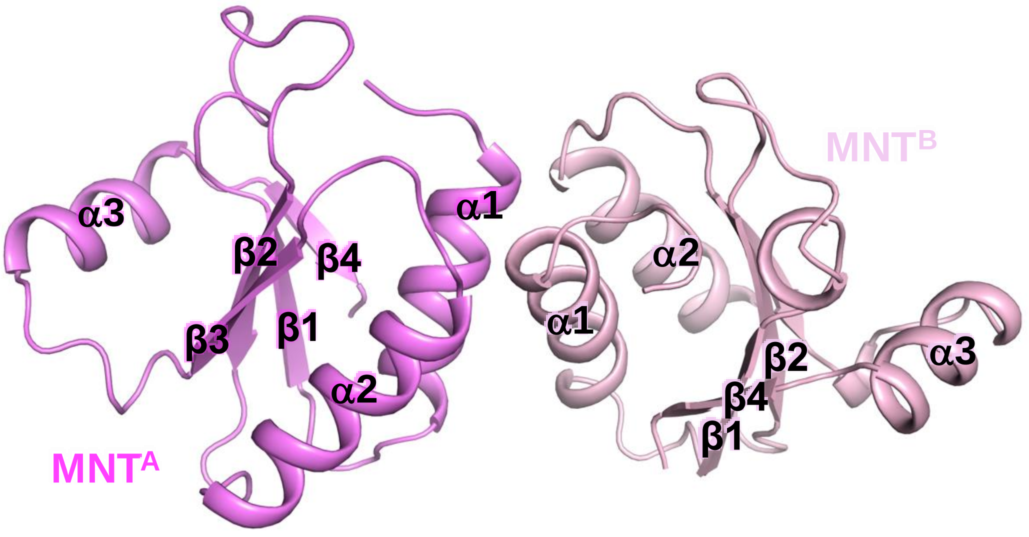

Supplementary Fig. 6. Overall structure of MNT^{Lpg}.

MNT^{Lpg} forms a homodimer in the asymmetric unit (ASU). Chains A and B of MNT are represented in violet and pink, respectively.

Supplementary Figure 7

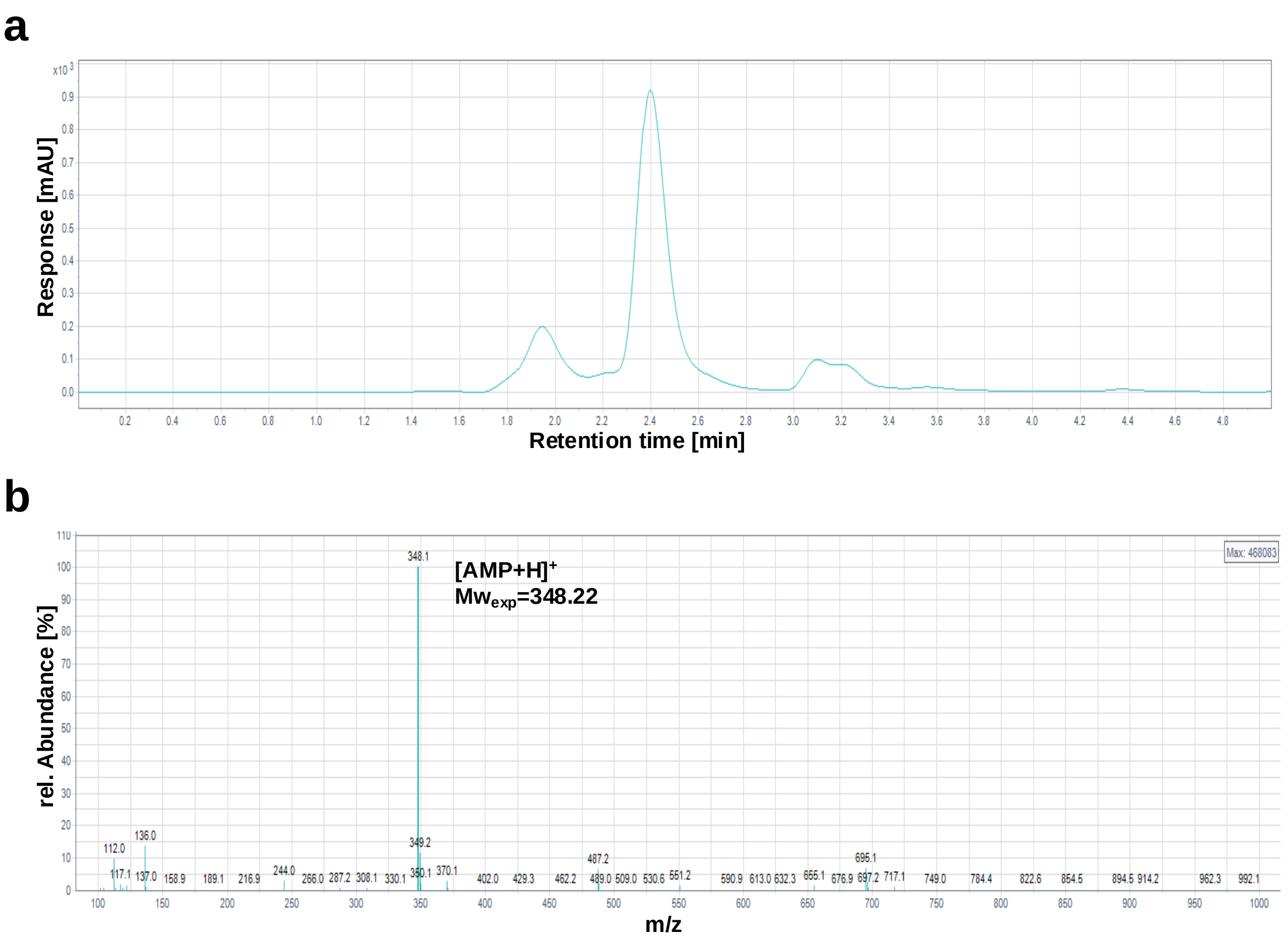

Supplementary Fig. 7. Analysis and characterization of NMPs digested from NMPylated HEPN^{Lpg} using LC–MS.

a The sample was separated by HPLC, and the peaks were measured and collected at 260 nm. **b** The separated sample was ionized, and the mass-to-charge ratio (m/z) was determined by an MS system. The obtained result revealed a peak at m/z 348.1, corresponding to the molecular weight of AMP.

Supplementary Figure 8

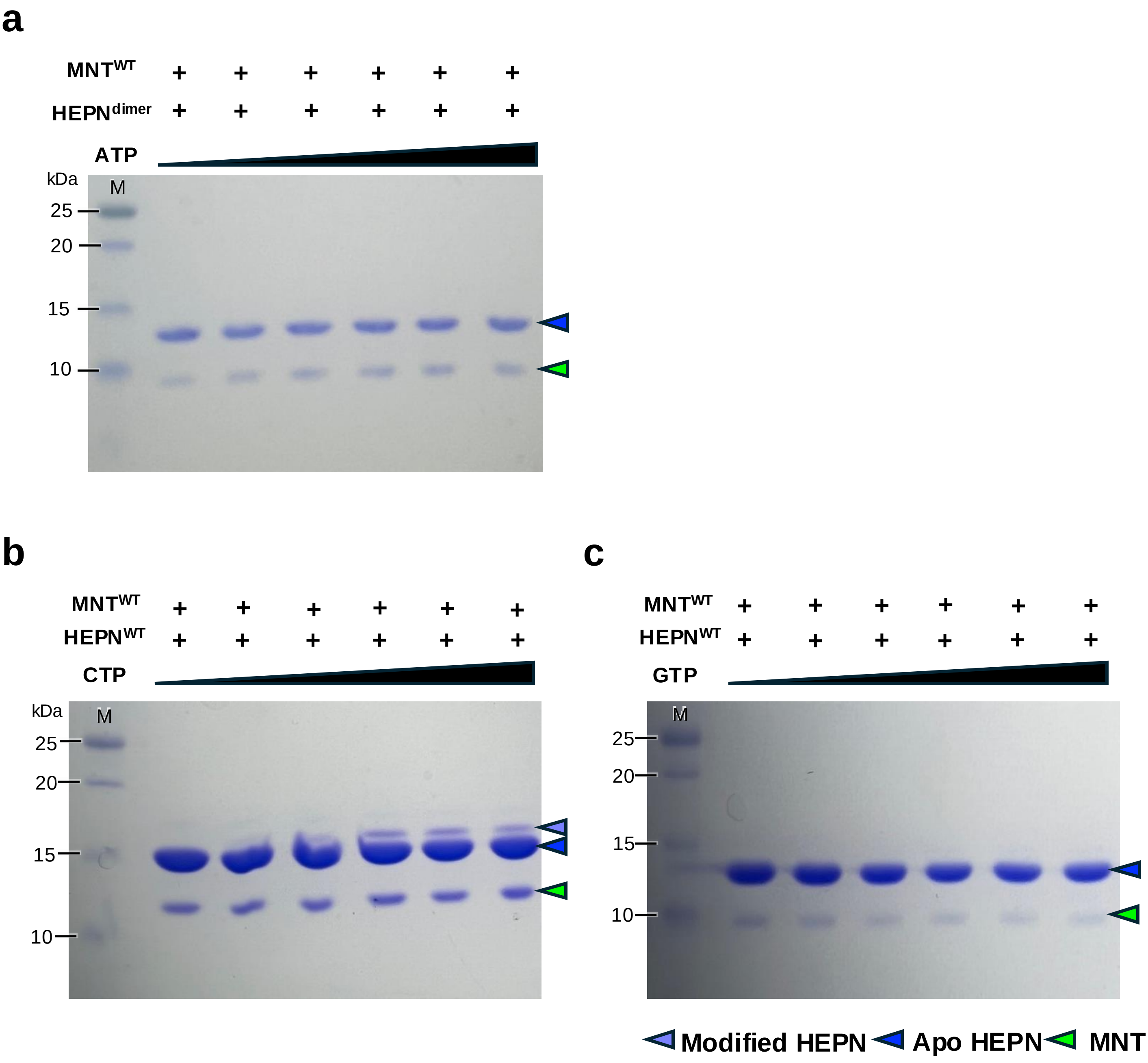

Supplementary Fig. 8. *In vitro* NMPylation assay.

a *In vitro* AMPylation assay conducted by dimeric HEPN^{Lpg} and MNT^{Lpg} WT

In vitro CMPylation assay (**b**) and *in vitro* GMPylation assay (**c**) were conducted by HEPN^{Lpg} WT and MNT^{Lpg} WT. All experiments involved increasing the substrate concentration.

Supplementary Figure 9

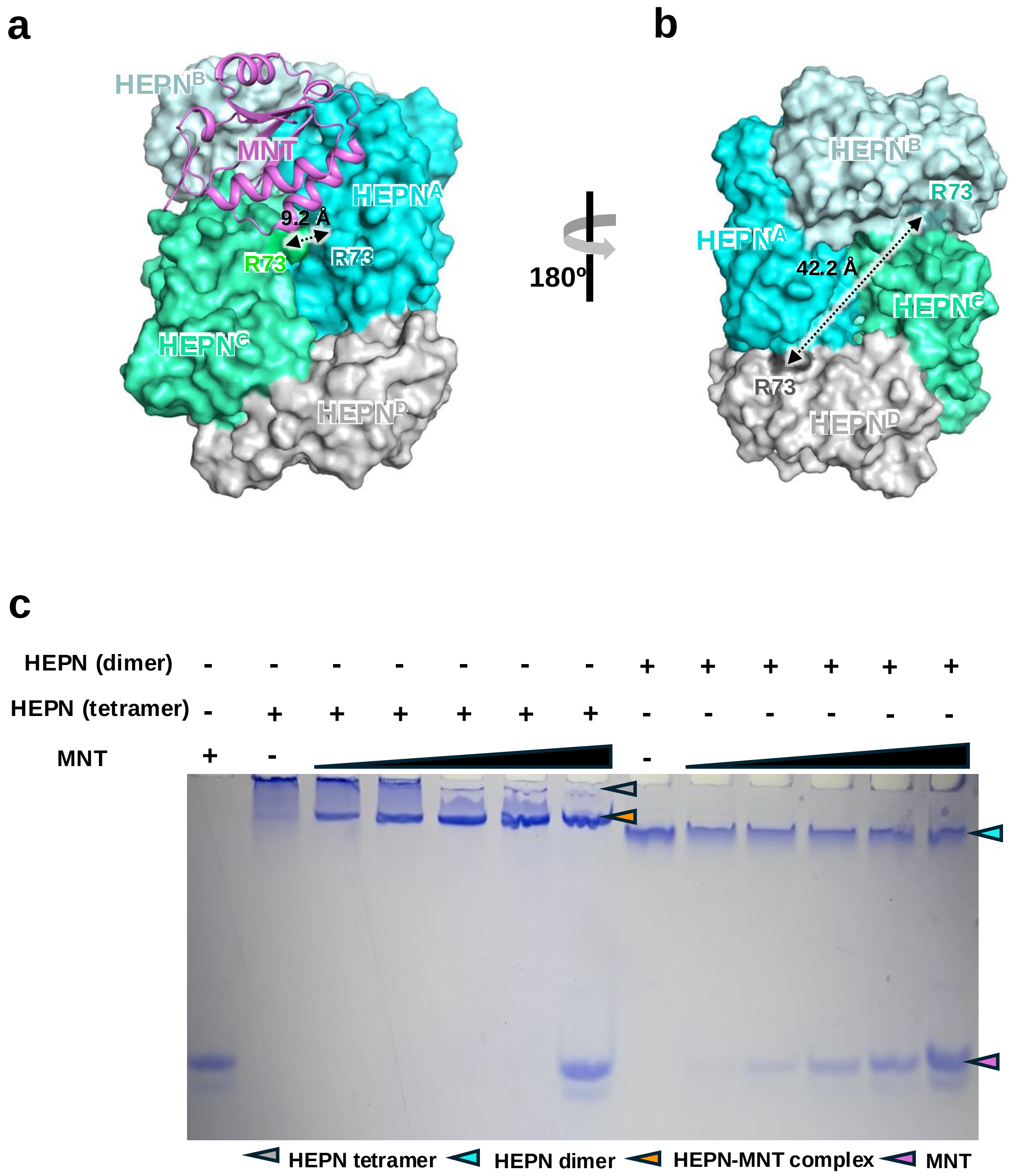

Supplementary Fig. 9. The binding stoichiometry between HEPN^{Lpg} and MNT^{Lpg}.

a, b Overall structure of the HEPN^{Lpg}–MNT^{Lpg} complex. The front (**a**) and opposite (**b**) perspectives aligning with the viewpoints presented in Fig. 8a, b. The distance between R73 residues in chains A and C is 9.2 Å, while the distance between R73 residues in chains B and D is 42.2 Å. **c** Analysis of binding stoichiometry by Coomassie blue-stained native-PAGE. MNT^{Lpg} was subjected to incubation with dimeric or tetrameric HEPN^{Lpg} at the following ratios: 1:5, 1:2, 1:1, 2:1 and 5:1. HEPN^{Lpg} Q64A was used as dimeric form of HEPN^{Lpg}. The formation of a HEPN^{Lpg}–MNT^{Lpg} complex occurred exclusively when MNT^{Lpg} was introduced to tetrameric HEPN^{Lpg}.

Supplementary Figure 10

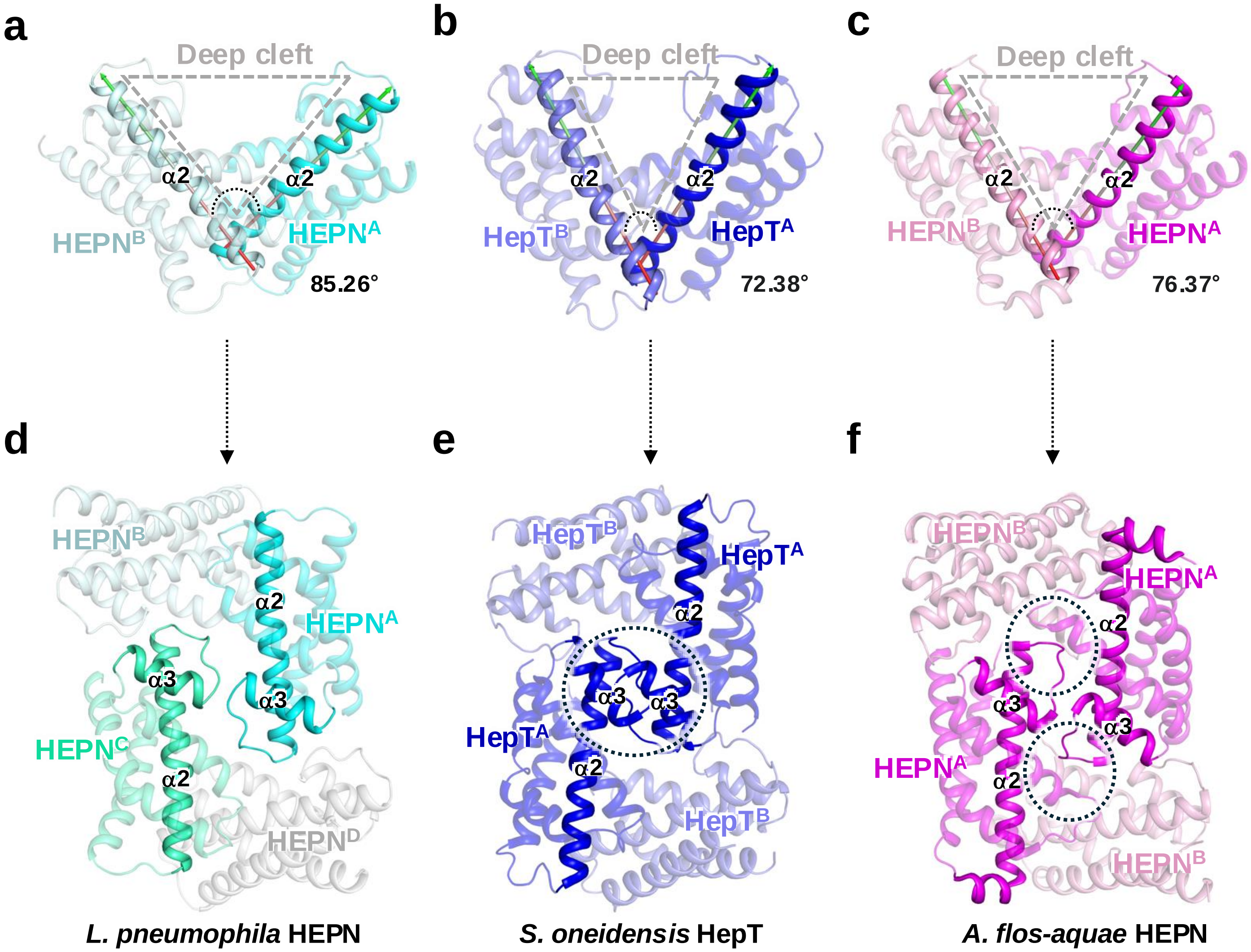

Supplementary Fig. 10. Molecular basis of oligomeric state transition.

Dimeric state of HEPN from *L. pneumophila* (**a**), *S. oneidensis* (**b**) and *A. flos-aqua* (**c**). The deep cleft region is indicated by a dashed triangle, and the angle between the α2 helix and each monomer was calculated by *PyMOL*. **d** Crystal structure of $HEPN^{Lpg}$ WT; **e** Tetrameric HepT model from *S. oneidensis*; **f** Tetrameric HEPN model from *A. flos-aquae*. The tetrameric structures of HepT from *S. oneidensis* and HEPN from *A. flos-aquae* were constructed using the crystal structure of $HEPN^{Lpg}$ WT as a model. The α2 and α3 helices involved in tetrameric interfaces are highlighted. Due to the constricted space within a deep cleft, a steric clash would occur upon the assembly of *S. oneidensis* HepT and *A. flos-aquae* HEPN into a tetramer. The specific clash sites are indicated by black dashed circles.

Supplementary Figure 11

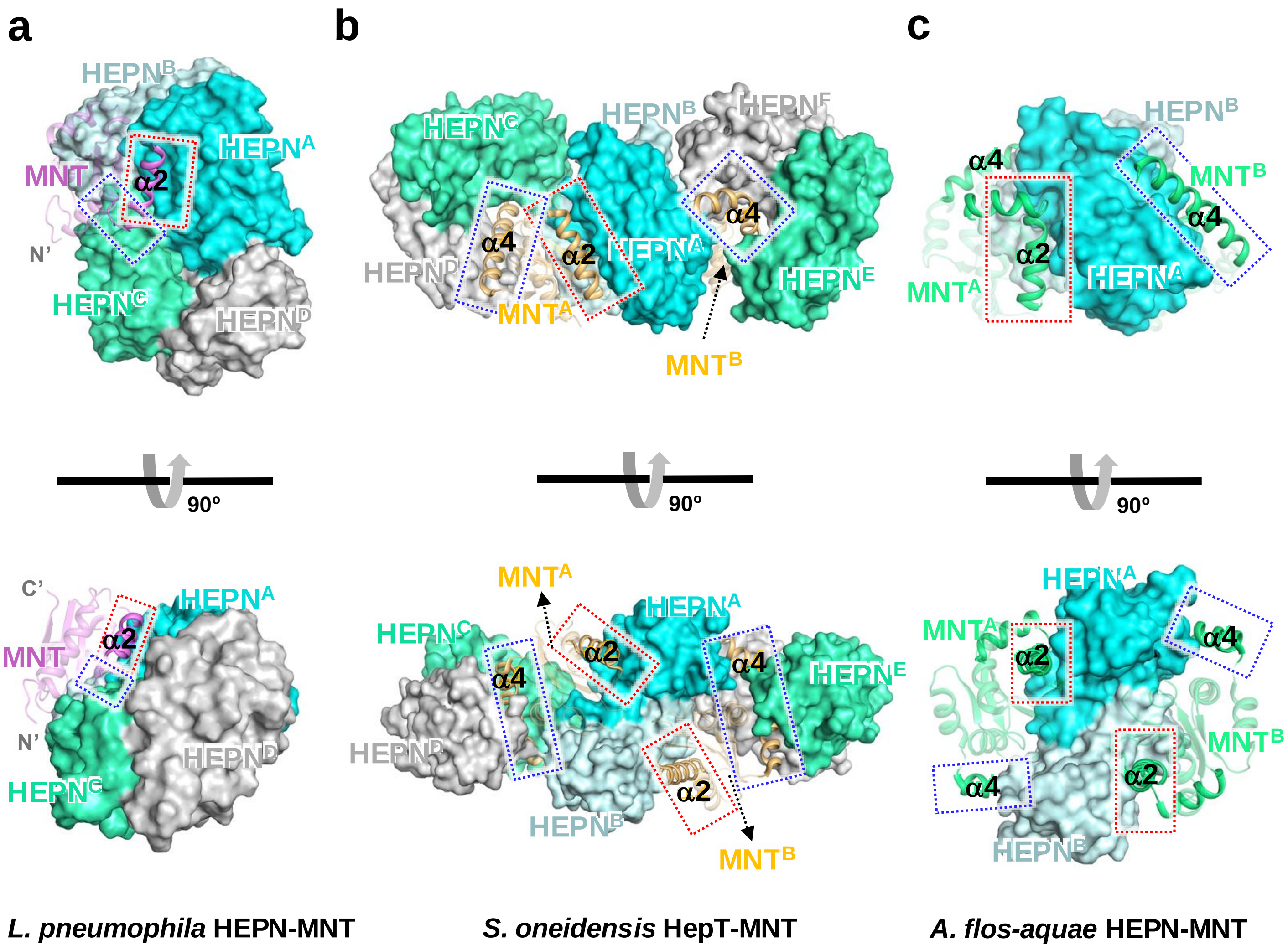

Supplementary Fig. 11. Comparison of the HEPN^{Lpg}-MNT^{Lpg} complex with its homologs.

a The HEPN–MNT complex from *L. pneumophila*. **b** The HEPN–MNT complex from *S. oneidensis* (PDB ID: 7BXO). **c** The HEPN–MNT complex from *A. flos-aqua* (PDB ID: 7AE2). MNTs and HEPNs are depicted as cartoons and surfaces, respectively. The α2 helices in MNT from all three homologs participate in interacting with HEPN, establishing a "primary docking site". Additionally, the α4 helices in MNT from *S. oneidensis* and *A. flos-aqua* contribute to a "secondary docking site", whereas in MNT^{Lpg}, a loop region following the α2 helix is implicated in this site. The primary docking site and secondary docking site are indicated by red and blue dashed squares, respectively.

Supplementary Table 1. Surface area of the crystal structures of the HEPN^{Lpg}–MNT^{Lpg} module

**Type of interface**	**HEPN^{Lpg} WT**	**Surface area (Å^2)**		**Entire surface area (Å^2)**	**Buried surface Area (Å^2)**	**Buried surface area percentage (%)**
Dimeric	Chains A/B	Chain A: 7214	Chain B: 7339	14553	882	6.1
	Chains C/D	Chain C: 7121	Chain D: 7265	14386	874	6.1
Tetrameric	Chains A/D	Chain A: 7214	Chain D: 7265	14479	405	2.8
	Chains B/C	Chain B: 7339	Chain C: 7121	14460	419	2.9
	Chains A/C	Chain A: 7214	Chain C: 7121	14335	311	2.2
**Type of interface**	**HEPN^{Lpg} Q64A**	**Surface area (Å^2)**		**Entire surface area (Å^2)**	**Buried surface Area (Å^2)**	**Buried surface area percentage (%)**
Dimeric	Chains A/B	Chain A: 7490	Chain B: 7607	15097	928	6.1
**Type of interface**	**HEPN^{Lpg}–MNT^{Lpg} complex**	**Surface area (Å^2)**		**Entire surface area (Å^2)**	**Buried surface Area (Å^2)**	**Buried surface area percentage (%)**
Dimeric	Chains A/B	Chain A: 7744	Chain B: 7167	14911	848	5.7
	Chains C/D	Chain C: 7438	Chain D: 6941	14379	975	6.8
Tetrameric	Chains A/D	Chain A: 7744	Chain D: 6941	14685	430	2.9
	Chains B/C	Chain B: 7167	Chain C: 7438	14605	434	3.0
	Chains A/C	Chain A: 7744	Chain C: 7438	15182	273	1.8
HEPN–MNT complex	Chians A/E	Chain A: 7744	Chain E: 6224	13968	512	3.7
	Chains C/E	Chain C: 7438	Chain E: 6224	13662	629	4.6

Supplementary Table 2. Relative RNase activity of dimeric HEPN^{Lpg} and its variants

HEPN^{Lpg}	V_{max}	k_{cat}[a]	K_m[a]	k_{cat}/K_m
**Dimeric HEPN^{Lpg}**	27.18	271.80	0.69	393.91
**Dimeric HEPN^{Lpg}+EDTA**	0.93	9.34	0.80	11.68
**Dimeric HEPN^{Lpg} Q44A**	1.99	19.88	0.92	21.61
**Dimeric HEPN^{Lpg} E47A**	1.69	16.86	1.01	16.69
**Dimeric HEPN^{Lpg} Q44AE47A**	0.98	9.83	0.95	10.35

[a]The units for k_{cat} and K_m are defined as RFU min^{-1} μM^{-1} and μM, respectively.

Supplementary Table 3. Oligonucleotides used in the study

Oligomer	Sequence
HEPN^{Lpg}-F	5′-GGA ATT CCA TAT GAC CAA TAT CGA TGT TCG C-3′
HEPN^{Lpg}-R	5′-CCG CTCGAG TTA TTC ATG CTC TTT CAG ACC-3′
MNT^{Lpg}-F	5′-GGA ATT CCA TAT GAA TGA GCA ATT AAA CC-3′
MNT^{Lpg}-R	5′-CCG CTCGAG TTA CGA TTC TTT TGT ATA AAA C-3′
HEPN^{Lpg}-MNT^{Lpg}-F	5′-GGA ATT CCA TAT GAC CAA TAT CGA TGT TCG C-3′
HEPN^{Lpg}-MNT^{Lpg}-R	5′-CCG CTC GAG TTA CGA TTC TTT TGT ATA AAA CAG G-3′
HEPN^{Lpg} R98A-F	5′-GGA TGG AAA TGA TTA AAA GTG CCA ACC AAA CCT CTC ATA CCT A-3′
HEPN^{Lpg} R98A-R	5′-TAG GTA TGA GAG GTT TGG TTG GCA CTT TTA ATC ATT TCC ATC C-3′
HEPN^{Lpg} H103A-F	5′-AAA AGT CGC AAC CAA ACC TCT GCT ACC TAC AAC CAA TCC GTT GCC-3′
HEPN^{Lpg} H103A-R	5′-GGC AAC GGA TTG GTT GTA GGT AGC AGA GGT TTG GTT GCG ACT TTT-3′
HEPN^{Lpg} Q44A-F	5′-GGA AAA GCA AGG CTT AAT CGC AGC CTT TGA ATT TAC CCA TG-3′
HEPN^{Lpg} Q44A-R	5′-CAT GGG TAA ATT CAA AGG CTG CGA TTA AGC CTT GCT TTT CC-3′
HEPN^{Lpg} E47A-F	5′-CTT AAT CCA AGC CTT TGC ATT TAC CCA TGA GCT GG-3′
HEPN^{Lpg} E47A-R	5′-CCA GCT CAT GGG TAA ATG CAA AGG CTT GGA TTA AG-3′
HEPN^{Lpg} Q44AE47A-F	5′-AAA GCA AGG CTT AAT CGC AGC CTT TGC ATT TAC CCA TGA GCT GG-3′
HEPN^{Lpg} Q44AE47A-R	5′-CCA GCT CAT GGG TAA ATG CAA AGG CTG CGA TTA AGC CTT GCT TT-3′
HEPN^{Lpg} Q64A-F	5′-TGA AAG ATT ATT TCT TTT TCG CGG GAA ATT CTG CAA TTA CTG G-3′
HEPN^{Lpg} Q64A-R	5′-CCA GTA ATT GCA GAA TTT CCC GCG AAA AAG AAA TAA TCT TTC A-3′
HEPN^{Lpg} R73A-F	5′-CTG CAA TTA CTG GTT CTG CTG ATG CAA CAC GCG AAT C-3′
HEPN^{Lpg} R73A-R	5′-GAT TCG CGT GTT GCA TCA GCA GAA CCA GTA ATT GCA G-3′
MNT^{Lpg} G36AS37T-F	5′-TGA TAA TGC GAT TCT CTA CGC AAC TCG TGC CAA AGG CAC ATA TC-3′
MNT^{Lpg} G36AS37T-R	5′-GAT ATG TGC CTT TGG CAC GAG TTG CGT AGA GAA TCG CAT TAT CA-3′
MNT^{Lpg} D48E-F	5′-GGC ACA TAT CAT CAG GGC TCA GAG ATT GAT CTT TGC CTT ACC GGA-3′

MNT[Lpg] D48E-R	5′-TCC GGT AAG GCA AAG ATC AAT CTC TGA GCC CTG ATG ATA TGT GCC-3′
MNT[Lpg] D50E-F	5′-CAT CAG GGC TCA GAT ATT GAG CTT TGC CTT ACC GGA AAC-3′
MNT[Lpg] D50E-R	5′-GTT TCC GGT AAG GCA AAG CTC AAT ATC TGA GCC CTG ATG-3′
MNT[Lpg] D48ED50E-F	5′-CAT ATC ATC AGG GCT CAG AGA TTG AGC TTT GCC TTA CCG GAA A-3′
MNT[Lpg] D48ED50E-R	5′-TTT CCG GTA AGG CAA AGC TCA ATC TCT GAG CCC TGA TGA TAT G-3′

Supplementary Table 4. Statistics for data collection and model refinement

Dataset	MNT^{Lpg}	$HEPN^{Lpg}$ WT	$HEPN^{Lpg}$ Q64A	AMPylated $HEPN^{Lpg}$	$HEPN^{Lpg}$-MNT^{Lpg} complex
**Data collection**					
Wavelength (Å)	0.97949	0.97942	1.02000	0.97933	0.97942
Space group	$P\,2_12_12_1$	$C\,2$	$P\,222$	$C\,2$	$C\,2$
Cell dimensions					
a, b, c (Å)	58.17, 61.59, 66.48	143.88, 53.19, 86.97	43.774, 43.772, 163.302	144.199, 53.296, 88.021	113.563, 93.747, 84.958
α, β, γ (°)	90.00, 90.00, 90.00	90.00, 122.10, 90.00	90.00, 90.00, 90.00	90.00, 122.31, 90.00	90.00, 123.86, 90.00
Resolution range (Å)	50.00–2.79 (2.85–2.79)	50.00–1.80 (1.83–1.80)	50.00–1.59 (1.69–1.59)	50.00–1.65 (1.68–1.65)	50.00–2.40 (2.44–2.40)
Total/unique reflections	37 577/6 291	345 212/51 227	273 546/67 303	444 697/67 341	195 822/28 576
Completeness (%)	99.7 (99.7)	98.4 (99.2)	83.0 (65.3)	99.6 (99.5)	98.4 (98.1)
Redundancy	6.0 (5.2)	6.7 (6.7)	4.1 (3.2)	6.6 (6.3)	6.9 (6.7)
R_{merge} (%)[a]	13.0 (53.6)	10.2 (58.7)	6.5 (37.7)	12.6 (229.3)	10.3 (56.0)
Mean I/σ (I)	22.3 (2.4)	24.8 (2.7)	11.8 (1.9)	35.4 (2.3)	20.3 (2.0)
$CC_{1/2}$	0.974 (0.967)	0.994 (0.881)	0.997 (0.862)	0.996 (0.662)	0.996 (0.847)
**Refinement**					
Resolution range (Å)	45.18–2.79	48.75–1.80	42.28–1.59	29.01–1.65	47.15–2.40
R_{work}/R_{free}[b]	23.1/28.7	16.4/20.4	18.8/21.4	17.8/20.8	19.8/24.7
RMSDs					
Bond lengths (Å)	0.002	0.006	0.011	0.013	0.002
Bond angles (°)	0.451	0.913	0.843	1.264	0.489
Number of atoms/average B-factors (Å^2)					
Protein	1528/62.96	4342/30.48	2198/24.24	4342/29.03	5104/50.49
Water	26/54.93	457/39.86	306/34.11	394/40.51	141/42.44
Other	-	2/39.36	1/22.24	90/71.54	-
Ramachandran plot					
Most favored (%)	95.79	98.84	99.24	99.03	97.88
Allowed (%)	4.21	1.16	0.76	0.97	2.12
Outliers (%)	0.00	0.00	0.00	0.00	0.00
Rotamer outliers	0.60	0.00	0.43	0.00	0.91
PDB accession code	8XEO	8XEM	8YUF	8XDJ	8XEH

[a] $R_{merge} = \Sigma_h \Sigma_i \mid I(h)_i - < I(h) > \mid / \Sigma_h \Sigma_i I(h)_i$, where $I(h)$ is the intensity of reflection h, Σ_h is the sum over all reflections, and Σ_i is the sum over i measurements of reflection h.

[b] $R = \Sigma \mid |F_{obs}| - |F_{calc}| \mid / \Sigma |F_{obs}|$, where R_{free} is calculated for a randomly chosen 5% of reflections, which were not used for structure refinement and R_{work} is calculated for the remaining reflections.
