# Supplementary material for: Structural insight into the distinct regulatory mechanism of the HEPN–MNT toxin-antitoxin system in Legionella pneumophila
Source: Nat Commun. 2024 Nov 24;15:10188. doi: 10.1038/s41467-024-54551-0 (PMC11586414; doi:10.1038/s41467-024-54551-0)
Supplement: Supplementary file 2 — Description of Additional Supplementary Files [file 41467_2024_54551_MOESM2_ESM.pdf]

Description of Additional Supplementary Files

File name: Supplementary Data 1
Description: MD simulations input model and output files and MD simulations reliability and reproducibility checklist.

File name: Supplementary Data 2
Description: LC-MS raw data files.

File name: Supplementary Data 3
Description: High-resolution LC-MS/MS raw data for apo HEPNLpg and NMPylated-HEPNLpg.
