# Supplementary material for: Structural insight into the distinct regulatory mechanism of the HEPN–MNT toxin-antitoxin system in Legionella pneumophila
Source: Nat Commun. 2024 Nov 24;15:10188. doi: 10.1038/s41467-024-54551-0 (PMC11586414; doi:10.1038/s41467-024-54551-0)
Supplement: Supplementary file 3 — Supplementary Data 1 [file 41467_2024_54551_MOESM3_ESM.zip › Supplementary Data 1/md-simulation-checklist_Final.docx]

| **Reliability and reproducibility checklist for molecular dynamics simulations**  ***All boxes must be marked YES by acceptance unless an N/A option is available** | | | **Yes** | **N/A** | **Response  (Please state where this information can be found in the text)** |
| --- | --- | --- | --- | --- | --- |
| **1. Convergence of simulations and analysis** | | | | | |
| 1a. Is an evaluation presented in the text to show that the property being measured has equilibrated in the simulations (*e.g.* time-course analysis)? | | |  |  | In the methods section, we describe the evaluation of equilibration for the properties measured during the simulations. The solvated system containing the protein underwent energy minimization and relaxation for 100 ps using the minimization step in Desmond with the OPS2005 forcefield. Following this, the simulations were conducted in the NPT ensemble (isothermal and isobaric) using the Martyna-Tobias-Klein method for isotropic pressure at 1 atm, and the Nose-Hoover thermostat algorithm to maintain a constant temperature of 300K. |
| 1b. Then, is it described in the text how simulations are split into equilibration and production runs and how much data were analyzed from production runs? | | |  |  | In the methods section, we describe how the simulations were divided into equilibration and production runs. The equilibration phase lasted for 100 ps, during which the system was relaxed. Following this, a total of 200 ns of production simulations were performed, with trajectories saved at 200 ps intervals. The trajectories from the production runs were analyzed using the simulation interaction diagram. |
| 1c. Are there at least 3 simulations per simulation condition with statistical analysis? | | |  |  | For each simulation condition, we conducted at least three independent simulations. |
| 1d. Is evidence provided in the text that the simulation results presented are independent of initial configuration? | | |  |  | We conducted multiple simulations starting from different initial configurations and analyzed the resulting properties. The results showed consistent behavior across these different configurations, indicating that the outcomes are robust and not significantly influenced by the initial setup. |
| **2. Connection to experiments** | | | | | |
| 2a. Are calculations provided that can connect to experiments (*e.g.* loss or gain in function from mutagenesis, binding assays, NMR chemical shifts, J-couplings, SAXS curves, interaction distances or FRET distances, structure factors, diffusion coefficients, bulk modulus and other mechanical properties, *etc*.)? | | |  |  | In our study, we analyzed the kinetic and dynamic movements of each residue by comparing the RMSF graphs from our Molecular Dynamics results. This analysis provides insights into the stability and flexibility of residues, which can be correlated with experimental data from mutagenesis studies. |
| **3. Method choice** | | | | | |
| 3a. Is it described in the text what force field and water model are used and why? | | |  |  | Yes, it is described in the method sections. OPLS4 force field and TIP3P water models are used as highly accurate, modern force fields with comprehensive coverage of chemical space. |
| 3b. Do simulations contain membranes, membrane proteins, intrinsically disordered proteins, glycans, nucleic acids, polymers, or cryptic ligand binding? | | |  |  | Response not needed if **N/A** |
|  | If 3b is **YES**, are enhanced sampling methods used? | |  |  | Response not needed if **N/A** |
|  | | If enhanced sampling methods are used, are the convergence criteria clearly stated? |  |  |  |
|  | If 3b is **YES**, is it explained in the text why or why not enhanced sampling methods are used? | |  |  |  |
| **4. Code and reproducibility** | | | | | |
| 4a. Is a table provided describing the system setup, such as simulation box dimensions, total number of atoms, total number of water molecules, salt concentration, lipid composition (number of molecules and type)? | | |  |  | We described that information in the method section. Periodic boundary conditions with orthorhombic boxes buffered at 10x10x10 Å distances. The system was supplemented with 150 mM NaCl after being neutralized with sodium or chloride ions to maintain electrical balance.  However, the following information should be added:   \|  \| # of atoms \| # of water molecules \| \| --- \| --- \| --- \| \| HEPN_Apo \| 55372 \| 15565 \| \| HPEN_Q64A \| 34912 \| 10188 \| |
| 4b. Is it described in the text what simulation and analysis software and which versions are used? | | |  |  | Yes, it is described in the method. The Maestro software from the Schrödinger suite was employed for the molecular dynamics simulations. |
| 4c. Are initial coordinate and simulation input files and a coordinate file of the final output provided as supplementary files or in a public repository? | | |  |  | Yes, all relevant input files are included as supplementary data, along with the final output graphs and .dat files. |
| 4d. Is there custom code or custom force field parameters? | | |  |  | Response not needed if **N/A** |
|  | If **YES**, are they provided as supplementary profiles or in a public repository? | |  |  |  |
